# Cobalt ferrite magnetic nanoparticles as stirring actuators to improve UV–Vis spectroelectrochemical measurements in normal reflection mode

**DOI:** 10.1007/s00604-025-07351-2

**Published:** 2025-08-01

**Authors:** Alessandra Cutillo-Foraster, Nurhayat Özbek, Lluís Otero-de-Muller, Julio Bastos-Arrieta, Núria Serrano, José Manuel Díaz-Cruz

**Affiliations:** 1 Department of Chemical Engineering and Analytical Chemistry, University of Barcelona (UB), Martí I Franqués 1-11, 08028 Barcelona, Spain; 2 Department of Chemistry, Faculty of Sciences, Karadeniz Technical University, Trabzon, 61080 Turkey; 3 Water Research Institute (IdRA), University of Barcelona (UB), Martí I Franquès 1-11, 08028 Barcelona, Spain

**Keywords:** Spectroelectrochemistry (SEC), Fe(III)/Fe(II)-orthophenanthroline system, Screen-printed carbon electrodes (SPCE), Cobalt ferrite magnetic nanoparticles (CoFe_2_O_4_ MNPs), Nanostirring actuators, CTAB surfactant agent

## Abstract

Spectroelectrochemical (SEC) measurements using UV–Vis radiation in normal reflection mode (or normal configuration) are less sensitive than parallel approaches, since the volume of sample monitored has a much lower proportion of the diffusion layer created by the electrochemical processes, i.e., the region where relevant optical changes take place. In contrast, the normal configuration is more robust and reproducible and, as of today, is the only commercially available. This work presents a strategy to enhance normal reflection SEC measurements of Fe(III)/(II)-orthophenanthroline system using a screen-printed carbon electrode (SPCE), improving competitiveness with parallel designs. This method required the design of a new measuring cell based on the geometry of the commercial one, but replacing the eight magnets by a non-magnetic closing system. The developed approach involves adding cobalt ferrite magnetic nanoparticles (CoFe_2_O_4_ MNPs) to the analyte solution and coupling the SEC cell to a conventional magnetic stirrer. The resulting nanostirring, driven by MNPs movement, enhances mass transport toward the electrode. This accelerates diffusion layer renewal, leading to a noticeable increase of both electrochemical and optical signals.

**Graphical abstract:**

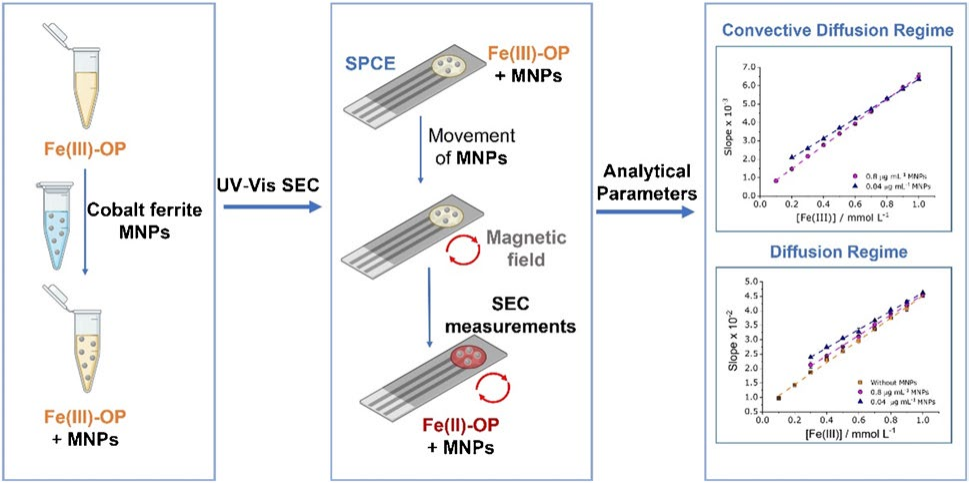

**Supplementary Information:**

The online version contains supplementary material available at 10.1007/s00604-025-07351-2.

## Introduction

Spectroelectrochemistry (SEC) is based on the combination of simultaneous electrochemical and spectroscopic measurements of substances, both electrochemically and optically active [1–5]. Although SEC has been mainly used for the characterization of materials and electrochemical reaction mechanisms, a few recent works point out interesting possibilities of SEC for analytical purposes, especially in the UV–Vis spectral region [6–11].

For many years, SEC measurements were restricted to optically transparent electrodes or thin-layer long-pathway cells but, recently, the increasing use of commercial screen-printed electrodes (SPE) [12–15] has provided a valuable tool to reinforce SEC possibilities for analytical purposes. In a previous work [11], different possibilities of quantification were achieved, including cathodic and anodic peaks in cyclic voltammetry (CV) and both electrochemical and optical signals acquired in chronoamperometric experiments.

Different configuration strategies that depend on the location of the optical beam relative to the electrode surface have been considered for enhancing SEC analysis. For instance, in the commonly used reflection mode (normal mode), the beam is placed perpendicularly to the electrode surface, making it suitable for miniaturized systems. Nevertheless, this configuration has lower sensitivity to parallel setup mode, in which the optical path crosses along the electrode surface, leading to higher interactions with analyte volumes that result in enhanced signal-to-noise ratios [16].

**Fig. 1:**
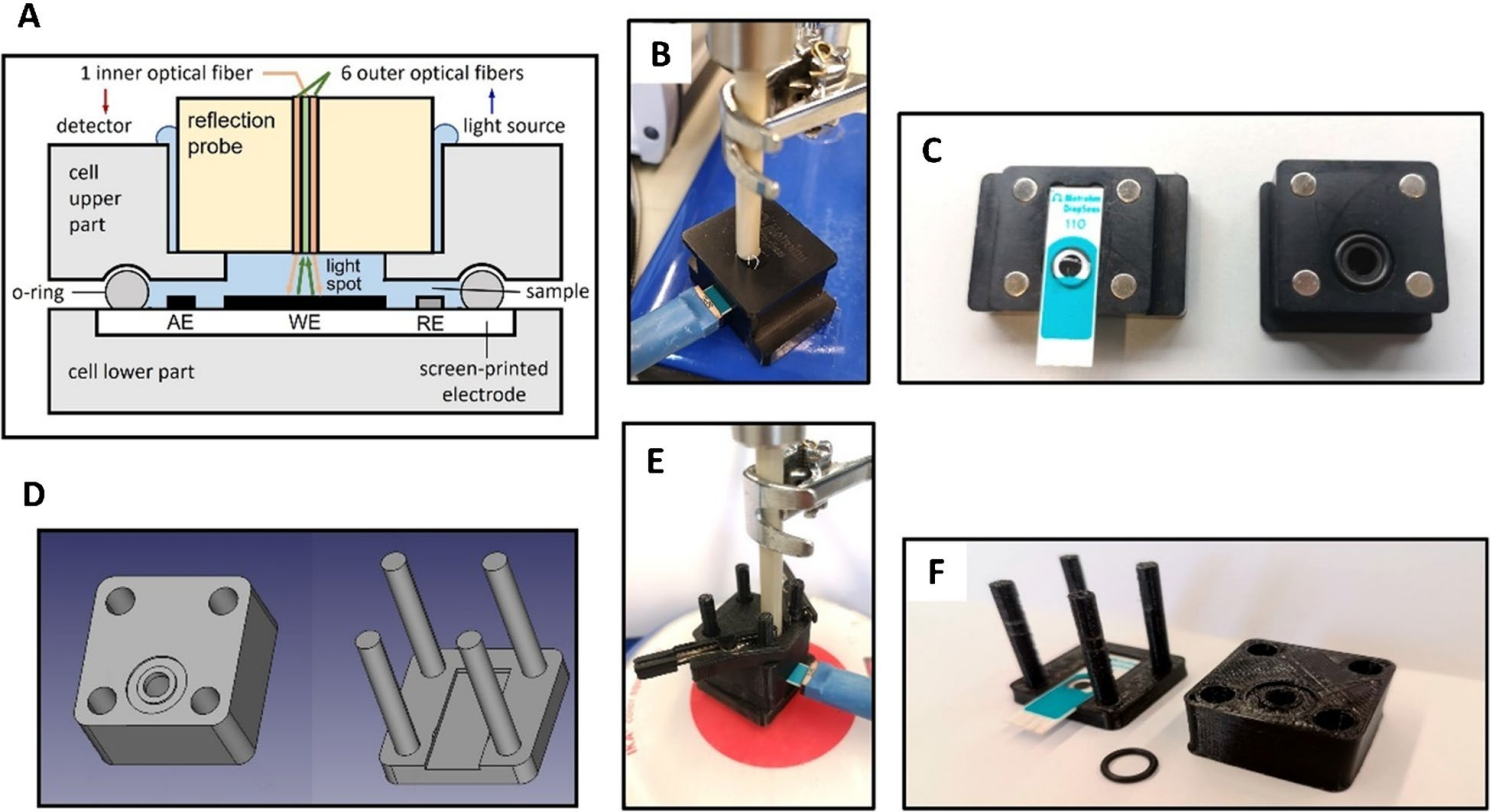
Cell setups used in this work for SEC measurements: **A** cross-section of the normal configuration, indicating the position of every element; **B** normal configuration with the reflection probe perpendicular inserted to the SPCE (with a CAST cable connector) in the commercial cell DRP-REFLECELL 70055; **C** view of the lower part of the commercial cell (left) with a SPCE inserted on it and 50 µL of solution covering all three electrodes, and the upper part (right) with the hole to contain the reflection probe and the o-ring to confine the sample when the cell is closed using the eight magnets shown in the image; **D** design of both parts of the magnet-free cell using FreeCAD software [35]; **E** new configuration substituting the commercial cell by a magnet-free cell, 3D-printed in PLA, and placing it on the center of a magnetic stirrer; and **F** view of the lower (left) and upper part (right) of the magnet-free cell, also showing the o-ring and an inserted SPCE unit

The previous work also evidenced that an important limitation to SEC sensitivity is the diffusion regime holding at the drop sample placed inside the measuring cell and in contact with the SPCE (Fig. 1A) [11]. This causes a fast depletion of the reactant near the electrode surface and a progressive broadening of the diffusion layer due to the slow mass transport from the bulk solution, governed by diffusion. Consequently, the measured current decreases very fast with time (Eq. 1) and a low sensitivity value is achieved. This could be improved by stirring the solution, in order to enhance the mass transport and transform the *diffusion regime* into *convective diffusion regime*, characterized by a constant width of the diffusion layer and a constant flow of both reagent and product [3]. This would result in a constant steady-state current and an absorbance proportional to time (since the integral of a constant *k* with respect to *t* is *kt*):

1 $$I= - k {c}_{\text{ox}}^{*}$$

2 $$A= k' {c}_{\text{ox}}^{*} t$$

being *k* and *k*′ constant values. Further details about SEC measurement considerations can be found in supplementary information: "Discussion on spectroelectrochemical measurements".

The incorporation of mass transfer enhancers, particularly magnetic nanoparticles (MNPs) acting as nanostirrers, is an interesting approach to overcome these limitations of normal reflection mode. MNPs have been extensively used for analytical purposes due to their excellent electromagnetic properties, low toxicity, adjustable size and shape, versatile coating, or modification methods [17, 18]. Typically, these nanomaterials have been used to selectively capture diverse analytes, combined with rapid magnetic separation and preconcentration of different analytes such as proteins, nucleic acids, and contaminants [19–23].

Active colloids are materials that can convert the energy or stimuli from their surroundings, such as chemical, magnetic field, or light ration, into kinetic energy [24]. Due to this self-propelled movement feature that provides efficient micromixing and enhanced mass transfer, different types of active colloids (e.g., catalytic micromotors) have been extensively applied in research fields, like electrochemical and optical (bio)sensing [25–27]. MNPs represent an active colloid-like material that can induce effective mixing and improved mass transfer in the nano/microscale [17, 18].

In this work, cobalt ferrite (CoFe_2_O_4_) MNPs will be used as stirring actuators in a confined and reduced volume (microliter scale) as an alternative approach to conventional stirring. This proof of concept is based on taking advantage of the propulsion of these cobalt ferrite MNPs in the measuring solution using a conventional magnetic stirrer to attain a convective diffusion regime near the electrode surface. CoFe_2_O_4_ MNPs present higher coercivity, magnetic saturation, chemical stability, and ease of functionalization compared to other ferrite (Fe₃O₄) MNPs [28–30]. All combined, these features provide enhanced magnetic responsiveness under rotating fields and stability in buffered aqueous solutions, useful for maintaining controlled, reproducible nanostirring in the proposed SEC system.

The active motion of MNPs under a rotating magnetic field to induce microscale convective flow remains an underexplored yet highly promising route for enhancing mass transport [31–33] in spectroelectrochemical setups. The nanostirring would induce the renewal of the solution near to the electrode surface, analogous to that resulting from conventional mechanical stirring or from constant solution flow and, consequently, an approach to a convective diffusion regime would be observed.

Therefore, the use of cobalt ferrite MNPs as nanostirrers to overcome diffusion limitations improves the analytical performance of SEC measurements in microliter-scale systems. The proposed nanostirred approach produces convective-diffusive mass transport in small-confined volumes (50 µL) without mechanical rotation or sophisticated microfabrication facilities [39, 40]. Our proposal makes SEC measurements more accessible and cost-effective.

## Experimental section

### Reagents

Analytical grade chemicals were used in all experiments. Sodium acetate trihydrate (CH_3_COONa·3H_2_O), acetic acid (glacial, 100%) (CH_3_COOH), and cetyltrimethylammonium bromide 99% (CTAB) were acquired from Merck (Darmstadt, Germany). The 1,10-phenanthroline monohydrochloride monohydrate 99% (OP·HCl·H_2_O) and ammonium iron(II) sulfate hexahydrate ((NH_4_)_2_Fe(SO_4_)_2_·6H_2_O) were purchased from Thermo Scientific (Waltham, USA). Stock solutions of Fe(III) were prepared by dilution from a standard iron solution of 1 mg mL^−1^ by Merck. Purified water (with electrical resistivity of 18.2 MΩ cm) was obtained from Milli-Q Reference A + Water Purification System (Millipore, France) to prepare all solutions. For the synthesis of CoFe_2_O_4_ MNPs, polyvinyloypyrrolidone (PVP) (Mw $\sim$ 29,000) and iron(III) nitrate nonahydrate (Fe(NO_3_)_3_·9H_2_O) were obtained from Merck and cobalt(II) nitrate hexahydrate 99% (Co(NO_3_)_2_·6H_2_O) was acquired from Thermo Scientific.

### Synthesis and characterization of cobalt ferrite magnetic nanoparticles

Cobalt ferrite magnetic nanoparticles (CoFe_2_O_4_ MNPs) were synthesized by modifying the procedure described by Naseri and colleagues [34], changing the use of nickel(II) salt to cobalt(II) salt precursor. Accordingly, for CoFe_2_O_4_ MNP synthesis, 3.5 g of PVP was dissolved in 100 mL of Milli-Q water at room temperature. Subsequently, 0.2 mmol of Fe(NO_3_)_3_·9H_2_O and 0.1 mmol of Co(NO_3_)_2_·6H_2_O (Fe:Co = 2:1) were introduced into the PVP solution and stirred for 2 h with a magnetic stirrer. Then, the solution was poured in a crystallizer and placed in an oven at 353 K for 24 h. An orange-brown solid was obtained, which was pulverized in a mortar to obtain an orange powder, which was calcinated at 823 K for 3 h to eliminate the organic compounds and crystallize the MNPs.

The characterization of synthesized MNPs was performed to verify the particle size and distribution using transmission electron microscopy (TEM) (JEOL JEM-1400), working at 120 kV as an accelerating voltage. TEM images were used to determine the morphology and particle size distribution. The surface charge of the cobalt ferrite MNPs using Zeta Potential was performed using a Malvern Zetasizer® Nano Z coupled with a red laser of − 10 mV. Magnetic measurements were carried out at the Unitat de Mesures Magnètiques of the Centres Científics i Tecnològics of the Universitat de Barcelona on a Quantum Design Susceptometer SQUID, MPMS XL7 Evercool. Hysteresis was measured between ± 7 T at 300 K.

### Study of cobalt ferrite MNP dispersions by optical microscopy

The behavior of CoFe_2_O_4_ MNPs in a suspension when a magnetic field is applied was observed using a stereomicroscope Euromex Stereoblue SB.1903-U (Duiven, The Netherlands) and pictures and videos were taken with Euromex VC.3031 HD Lite camera attached to the stereomicroscope. Specifically, this optical microscope was used for selecting the best conditions to minimize the agglomeration of MNPs under a magnetic field through comparing a solution without a surfactant agent and with different concentrations of this (0.1, 0.5, 1.0, and 5.0 mmol L^−1^). All the suspensions were prepared with acetate buffer solution (pH = 4.5). Moreover, it was observed with this technique the effect of these MNPs in the electrochemical process of Fe(III)/Fe(II)-OP system. Each stereomicroscope visualization was done in screen-printed silver electrodes (SPAgE) (reference DRP-010) provided by Metrohm-Dropsens (Oviedo, Spain) because this electrode provides a better visualization and higher contrast of the process that occur in the working electrode in comparison with SPCE in which the visual observation is difficult due to the black color of carbon as shown in Fig. S1. Moreover, to check that the electrochemical reaction happened, a cyclic voltammetry (CV) between − 0.3 and 0.0 V at 50 mV s^−1^ was performed.

### Spectroelectrochemical measurements

#### SEC measurements in normal reflection mode

SEC measurements were carried out using a SPELEC equipment from Metrohm-DropSens controlled by the software DropView (Version 3.2.2). Conventional SEC measurements were conducted in the commercial cell DRP-REFLECELL 70055, also by Metrohm-Dropsens (Oviedo, Spain), in normal configuration, i.e., with the SPE and the optical probe (RPROBE-VIS–NIR) placed horizontally and vertically, respectively (Fig. 1A to C). However, the closing system of this commercial cell is incompatible with the use of a magnetic stirrer needed for MNP experiments due to this cell is equipped with a closing mechanism based on eight magnets that firmly attach the two blocks of the device (Fig. 1C). To overcome this problem, a new magnet-free cell was specifically designed by the free software FreeCAD [35] and home-made fabricated in black polylactic acid (PLA) using the 3D printer XYZPrinter da Vinci 1.0 Pro. Figure 1D to F show the optimized version of the cell that was used in this work for nanostirring experiments (STL files available at: 10.34810/data2386).

Screen-printed carbon electrodes (SPCE) (reference DRP-110) were acquired from Metrohm-Dropsens, with both the working electrode (4-mm diameter) and the auxiliary electrode made of carbon, and with a silver pseudo-reference electrode. The SPCE was connected to SPELEC by means of a CAST cable connector, also by Metrohm-Dropsens. In some experiments, a light source DH-2000-BAL by Ocean Insight (Ostfildern, Germany) was used instead of that included in SPELEC in order to increase the radiation intensity, i.e., the flux of photons. The scattering effect caused by the surface of the working electrode of a SPCE unit is negligible because in each SEC measurement, a reference is set based on the initial conditions of the sample drop.

Non-containing MNPs samples are inserted into the commercial cell in five steps: (I) a SPCE unit is fixed onto the lower part of the cell; (II) the cell is closed by attaching the upper part, which remains fixed with eight magnets; (III) a volume of 100 μL of sample is added with a micropipette through the hole on the top of the cell; (IV) the optical probe is introduced in the hole and some drops are allowed to spill out from the sides of the probe; and (V) the CAST cable is connected to the SPCE. After each measurement, the cell and the SPCE are thoroughly washed with water and a fresh drop is used every time, to prevent substantial changes in the composition of the sample. As for SPCE, new electrodes are activated by immersing them in acetate buffer solution (the same used for the sample) and submitting them to four cycles (from 1.0 to − 0.4 V and then to 1.0 V). The activated SPCE units can be used for a series of 20–30 measurements and then are replaced with a new one.

In the SEC experiments carried out with the new design cell, the sample insertion procedure is essentially the same. The main difference is that the upper part of the cell is inserted by passing the four columns of the lower part through the holes in the upper part (Fig. 1D to F). The cell is properly placed in the center of a conventional magnetic stirrer to ensure a symmetric application of the magnetic field. In the present work, a big squid magnetic stirrer by IKA (Staufen, Germany) with a rotating disk-shaped magnet to produce a uniform rotating magnetic field was used at different rates up to 2000 rpm. The preliminary study was done using both cells (commercial and 3D cell). However, in the optimization and calibration curve, all SEC measurements were carried out using 3D printed cell.

The optical signal of SEC measurements was obtained in a wavelength range between 200 and 900 nm. As previously mentioned, the complex Fe(II)-OP generated in the reduction of Fe(III)-OP absorbs at a wavelength near 510 nm, which was confirmed by performing UV–Vis absorbance spectra of Fe(III)-OP and Fe(II)-OP complexes as shown in Fig. S2. The UV–Vis absorbance spectra were obtained using an Agilent Cary 60 UV–Vis spectrophotometer (CA, USA) for solutions containing 0.3 mmol L^−1^ of Fe(III) and 0.3 mmol L^−1^ of Fe(II) in 25 mmol L^−1^ of OP in acetate buffer solution (pH = 4.5). A solution with a concentration of 25 mmol L^−1^ of OP in acetate buffer solution was used as blank.

#### SEC measurements with CoFe_2_O_4_ MNPs as nanostirring actuators

All SEC measurements were conducted in solutions containing 25 mmol L^−1^ of OP in acetate buffer 10 mmol L^−1^ (pH = 4.5). Unless otherwise indicated, chronoamperometric measurements were carried out at a fixed potential of – 0.2 V every 0.5 s for 60 s. Integration time was set to 1000 ms when using the light source of SPELEC and to 100 ms with scan average of 10 spectra when using the light Source by Ocean, considering in both cases a set of 2007 wavelengths in the range 205–892 nm (that provided by the diode array detector of SPELEC).

The optimization of the operating conditions related to stirring rate was carried out in a solution containing 25 mmol L^−1^ of OP, 0.3 mmol L^−1^ Fe(III), and 0.8 µg mL^−1^ of cobalt ferrite MNPs at different stirring rates (0, 250, 500, 750, 1000, 1500, and 2000 rpm). Regarding the optimization of CoFe_2_O_4_ MNP concentration, this was performed in solutions with the same concentration of OP and Fe(III), mentioned previously, at 40, 8, 0.8, 0.08, and 0.04 µg mL^−1^ of CoFe_2_O_4_ MNPs.

#### Electrocatalytic effect of CoFe_2_O_4_ MNPs in the Fe(III)/Fe(II)-OP system

The evaluation of the electrocatalytic effect of CoFe_2_O_4_ MNPs in the Fe(III)/Fe(II)-OP system was carried out analyzing the CVs obtained between − 0.4 and 1.0 V at different scan rates (10, 20, 50, 100, and 200 mV s^−1^) for solutions without MNPs and with 40 and 0.8 µg mL^−1^ MNPs. All solutions were prepared in 10 mmol L^−1^ acetate buffer solution. These measurements were carried out with SPELEC equipment from Metrohm-DropSens controlled by the software DropView (Version 3.2.2).

#### Data treatment

Raw experimental data were imported from DropView software to Matlab® environment and processed with home-made programs written in Matlab [36]. Raw optical spectra in counts, acquired at 2007 wavelengths, were submitted to subtraction of the dark spectrum in the absence of radiation and compressed utilizing a four-level discrete wavelet transform (DWT) using Daubechies 4 function, which provided good results in previous studies with SPELEC [11, 37]. Absorbances were computed from DWT-compressed spectra in counts, using as a reference the corresponding spectra measured for the sample solution before the SEC measurement. The values of slopes of *A*_max_ obtained in Matlab were imported to Excel® to achieve a more precise data treatment of the experimental data.

Regarding data treatment of analytical parameters, the limit of detection (LOD) and limit of quantification (LOQ) were computed by using International Union of Pure and Applied Chemistry (IUPAC) definitions. Based on this, LOD and LOQ were calculated using 3*Sa*/*b* and 10*Sa*/*b* equations, respectively, where *Sa* corresponds to the standard deviation of the intercept and *b* is the slope of the calibration curve in the considered concentration. The linear range is comprised between the calculated LOQ and the maximum concentration at which linearity has been confirmed.

## Results and discussion

### Characterization of cobalt ferrite MNPs

**Fig. 2:**
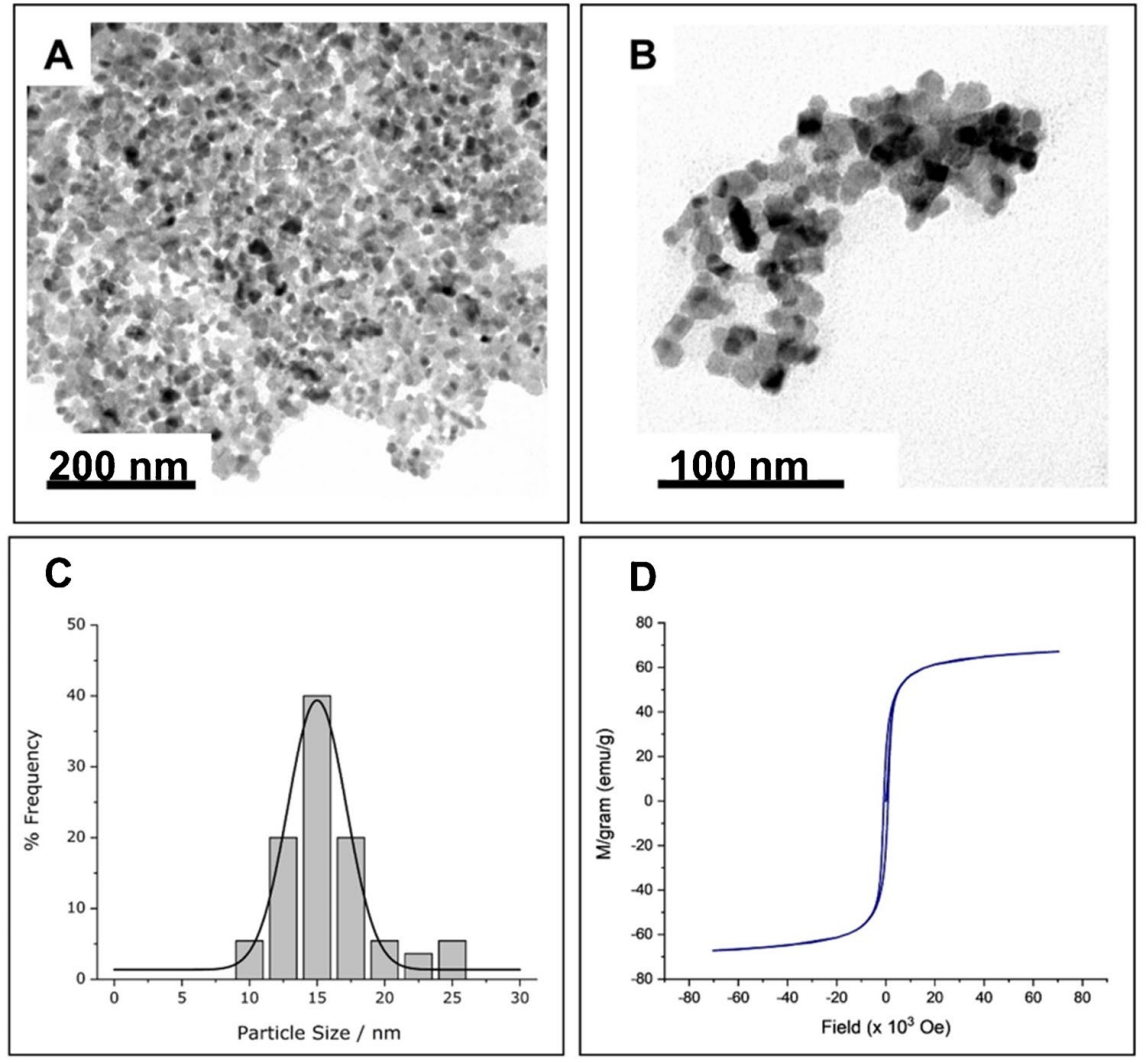
**A**, **B** TEM microphotographs at two different scales; **C** histogram of the size distribution of the synthesized cobalt ferrite MNPs; and **D** magnetization curves

TEM micrographs of CoFe_2_O_4_ MNPs, synthesized as described in the “Experimental section,” showed a quite sharp and symmetric size distribution (Fig. 2A to C), with an average diameter of 15 (± 3) nm. The zeta potential was − 10 mV, which indicates that their surface is slightly negatively charged, leading to possible agglomerate formation. The obtained MNPs presented a high magnetic moment (around 60 emu), which makes them suitable for their application as magnetic nanostirrers.

In order to verify the MNPs behavior during stirring stages, a stereomicroscope was used with a magnification from 7 × to 45 ×. The videos provided (Video S1 and S2) in the supplementary information summarize the results of such observation. It can be said that at high concentrations of MNPs (above 30 µg·mL^−1^, Video S1) multiple agglomerates of a large size are generated, even in the absence of magnetic stirring. The magnetostatic attraction between MNPs seems to be more important than the electrostatic repulsion due to their slightly negative charge. When the magnetic stirrer is on, the agglomerates show a clockwise rotation and a counter-clockwise translation, with increased speed for the particles that are far from the center of the electrode/magnetic stirrer. Moreover, raising the stirring rate causes an increase in both rotation and translation rates.

Optical microscopy observations revealed a change in the motion of MNPs aggregates as a function of stirrer speed. At low rotational speeds (below 250 rpm), aggregates predominantly exhibit localized rotation, while at higher speeds (500–750 rpm), translational displacement becomes increasingly evident. This alteration can be explained by the enhanced torque and hydrodynamic forces generated by the rotating magnetic field. A similar observation was reported in magnetically driven colloidal systems, where coordinated translation and rotation depend on field strength, the aggregate’s asymmetry, and hydrodynamic coupling [38–40]. At higher concentrations and magnetic field strengths, dipolar interactions among MNPs promote accumulation, possibly leading to partial magnetic, affecting both susceptibility and dynamic response [41, 42]. These observations explain the existence of an optimal operational window for nanostirring, compensating for convective enhancement with colloidal stability of the MNPs.

The average size of the agglomerates notoriously decreases by decreasing the concentration of MNPs. In order to avoid the formation of MNPs agglomerates, the cationic surfactant agent CTAB was added. As shown in Video S2, the addition of CTAB minimizes the formation of large agglomerations, which may be explained due to MNPs were stabilized in aqueous solution by decreasing their hydrophobic behavior through interaction between the non-polar chain of CTAB and MNPs surface [43]. Based on this observation for further experiments, a stock solution of cobalt ferrite MNPs was prepared by suspending them in acetate buffer solution (pH = 4.5) with 1 mmol L^−1^ of CTAB and from these, more diluted suspensions of MNPs were prepared.

### Preliminary study on the influence of MNPs on SEC measurements

It should be noted that in electrochemical cells containing a volume of solution in the order of some milliliters, stirring can be achieved by using a rotating electrode, a rotating bar, or a magnetic stirrer with a relatively small magnetic stir bar. Even in electrochemical flow cells working with solution volumes in the order of microliters, convective diffusion can be attained thanks to the continuous flow of the liquid (e.g., in amperometric detectors for liquid chromatography). However, in a confined volume of the order of 50–100 µL between the SPCE and the reflection probe, it is not possible to include a magnetic stir bar. Even in the case of using a very small stir bar, it would surely interfere with the UV–Vis beam of the probe. Taking all these concepts into consideration, in this work, cobalt ferrite MNPs were introduced as an alternative to conventional stirring. However, the movement of MNPs inside a few microliters of liquid could present many unexpected phenomena derived from the surface tension of the liquid, the reduced amount of analyte into the test solution that could cause a noticeable decrease of the total concentration if high currents are achieved, and the agglomeration of MNPs into bigger size particles able to produce dispersion or even blocking of the beam light of the optical probe.

The electrocatalytic effect of MNPs on SEC signals has been studied in the home-made cell by means of CV. For this purpose, 25 mmol L^−1^ of OP and 1.0 mmol L^−1^ of Fe(III) were used as sample solution, without and with 300 µg·mL^−1^ of MNPs. The corresponding results are summarized in Fig. S3.

Under these conditions, MNPs do not present specific absorption bands and only cause a slight decrease in the reflected counts (Fig. S3 A and B), probably due to light scattering induced by MNPs agglomerates. However, when the counts are normalized in the form of absorbances, the results are very similar (Fig. S3 C, D, and E). The same can be said regarding cyclic voltammograms (Fig. S3 F), which show the characteristic peaks of the oxidation of Fe(II) to Fe(III) at ca. 0.9 V and the reduction of Fe(III) to Fe(II) at ca. 0.2 V and are scarcely affected by the presence of cobalt ferrite MNPs. Furthermore, MNPs did not exhibit an electrocatalytic activity within the studied region (peak ca. 0.2 V of Fe(III) reduction) as shown in Fig. S3. These results confirm that the MNPs do not generate perturbations in SEC signals derived from their own electrochemical or optical activity.

An important exception to that is the formation of agglomerates of MNPs, which are visible with the naked eye and, eventually, may block the optical path, causing higher noise and a serious decrease in the spectra in counts. Fortunately, when this happens, the unusual shape of the spectrum is an effective warning to indicate that the measurement has to be repeated with a new drop of sample. Figure S5 compares a standard SEC measurement (in blue) with one containing MNPs (in red) without stirring. The chronoamperometric signals are very similar (Fig. S5 D), showing that the electrochemical process is not being affected. However, the considerably lower number of counts of spectra of the sample containing MNPs (Fig. S5 A) suggests that there is a significant loss of reflected radiation due to the above-mentioned dispersion by agglomerates. Also, there is an increased noise, more visible in the absorbance spectrum (Fig. S5 B).

Figure S5 also shows, in red color, the signals obtained when the magnetic stirrer is switched on. The overall shape of the spectrum in counts is not changing, but a larger variation of counts near the absorption maximum (ca. 510 nm) is detected (Fig. S5 A). This causes an absorbance higher than in the absence of magnetic stirring (Fig. S5 B and C). As for the current, it is also notoriously higher when the magnetic stirrer is working (Fig. S5 D). Thus, the increase in both the current and the absorbance suggests that MNPs are effectively moving with the external magnetic field and enhancing the mass transfer toward the electrode, which results in a higher production of the absorbing Fe(III)-OP complex. From Fig. S5 C and D, it is clear that such improvement of the mass transport is accompanied by an increase in the noise and some fluctuations in the current and the absorbance, probably caused by fluctuations in the movement of the MNPs. As for the precise mass transport regime, in the case of nanostirring, it should be close to convective diffusion, as the evolution of the maximum absorbance with time seems to approach linearity. The described MNPs behavior will be discussed in detail in the following sections.

**Fig. 3:**
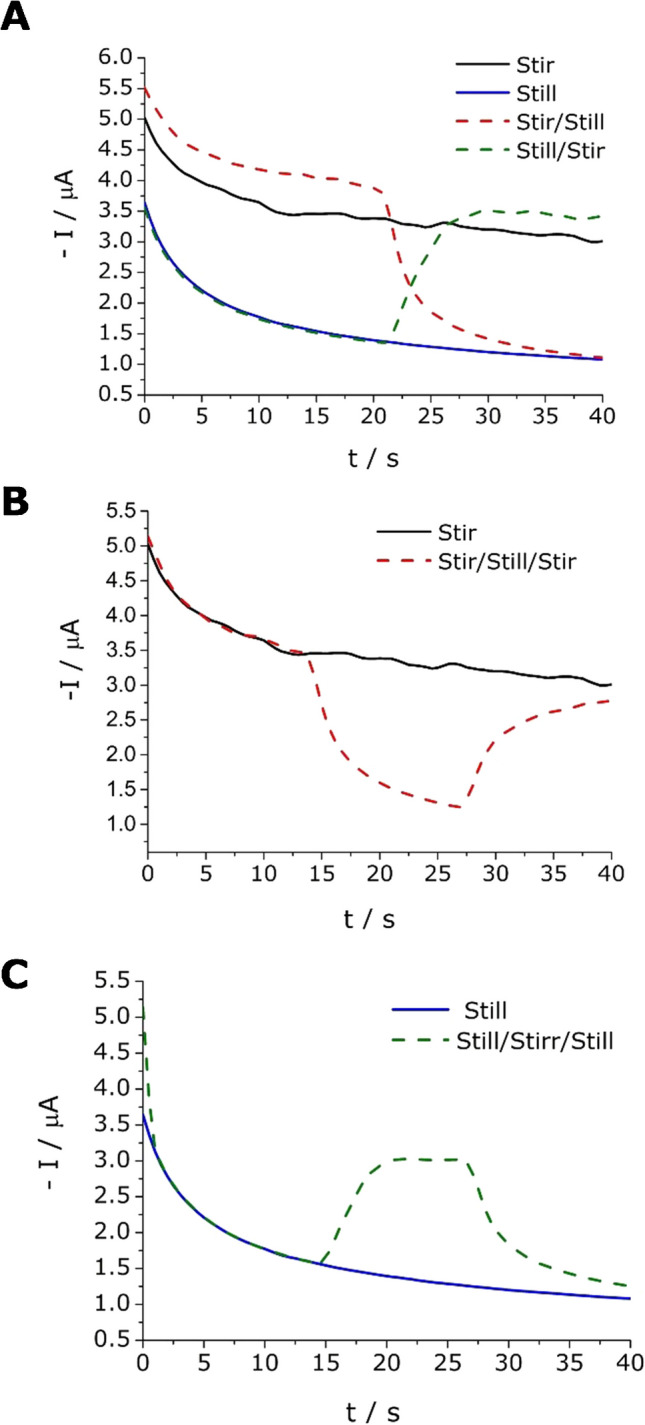
Effect of the application of a magnetic field in chronoamperometric measurements for a solution containing 25 mmol L^−1^ of OP and 0.4 mmol L^−1^ of Fe(III) in the presence of 400 µg mL^−1^ of MNPs at a fixed potential of − 0.2 V. Cathodic current as a function of time: **A** in the absence of stirring for 40 s (blue solid line); in the presence of stirring at 500 rpm during 40 s (solid black line); with 20 s of stirring at 500 rpm followed by 20 s without it (dashed red line); and with 20 s without stirring and 20 s with stirring at 500 rpm (dashed green line); **B** with 40 s of continuous stirring at 500 rpm (black solid line) and with 13 s of stirring at 500 rpm followed by 13 s without stirring and 13 s with stirring again (red dashed line); and **C** in the absence of stirring for 40 s (blue solid line) and without stirring for 13 s followed by 13 s of stirring at 500 rpm and 13 s without stirring (dashed green line)

To confirm this, a chronoamperometric assay, including stirring and static stages, was carried out and presented in Fig. 3. Under static conditions (without stirring), a fast decrease in the current is observed during the first 10 s and then a progressive stabilization is obtained (Fig. 3A, blue solid line). In contrast, the incorporation of a dynamic system through nanostirring with the addition of MNPs produces an important increase of the current, which, anyway, follows a similar pattern as before. This suggests an increase of mass transport from the bulk to the surface of the SPCE (Fig. 3A, solid black line). This trend could be modified by activating or deactivating the stirring stage, where the system changes from one to another (Fig. 3A, red and green dashed line). Moreover, it was observed that if a deactivation-activation-deactivation of the nanostirring is performed (Fig. 3C, green dash line), or the opposite process is done (Fig. 3B, red dash line), the system temporarily passes through its opposite and is capable of returning to the equilibrium of the original initial state. This behavior was independent of the initial measurement system (dynamic or stationary). These facts confirm that nanostirring enhances mass transport inside the measuring cell in a reversible way. Then, the next step is finding the conditions to take the maximum advantage of this effect.

### Optimization of the operating conditions for nanostirring in chronoamperometric SEC experiments

**Fig. 4:**
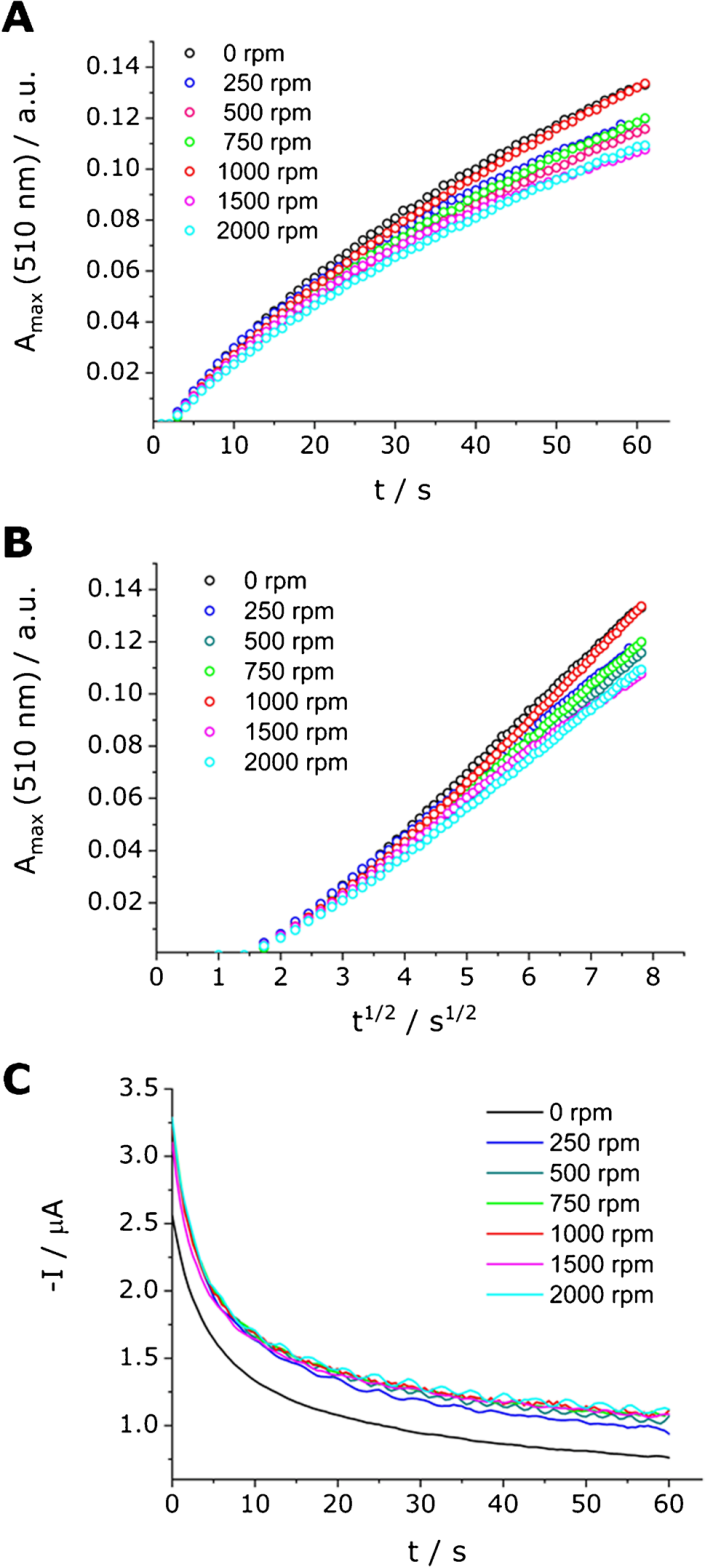
Effect of the stirring rate in UV–Vis SEC measurements for an OP concentration of 25 mmol L^−1^, Fe(III) concentration of 0.3 mmol L^−1^, and cobalt ferrite MNP concentration of 0.8 µg mL^−1^ at 0 (black), 250 (blue), 500 (dark cyan), 750 (green), 1000 (red), 1500 (pink), and 2000 (cyan) rpm at a fixed potential of − 0.2 V. **A** *A*_max_ (510 nm) as a function of *t*, **B** *A*_max_ (510 nm) as a function of *t*^1/2^, and **C** –*I* as a function of *t*

The key factors considered are the concentration of added MNPs and the rotation rate of the magnetic stirrer. First of all, the optimization of the stirring rate was conducted. As shown in Fig. 4A and B, the stirring velocity of 1000 rpm (red dots) displayed an optical response equal to the solution without MNPs (black dots). Regarding the electrochemical signal (Fig. 4C), all stirring velocities produce an increase in the current in chronoamperometric experiments compared to the solution without MNPs. Then, in further experiments with MNPs, a stirring rate of 1000 rpm was fixed.

**Fig. 5:**
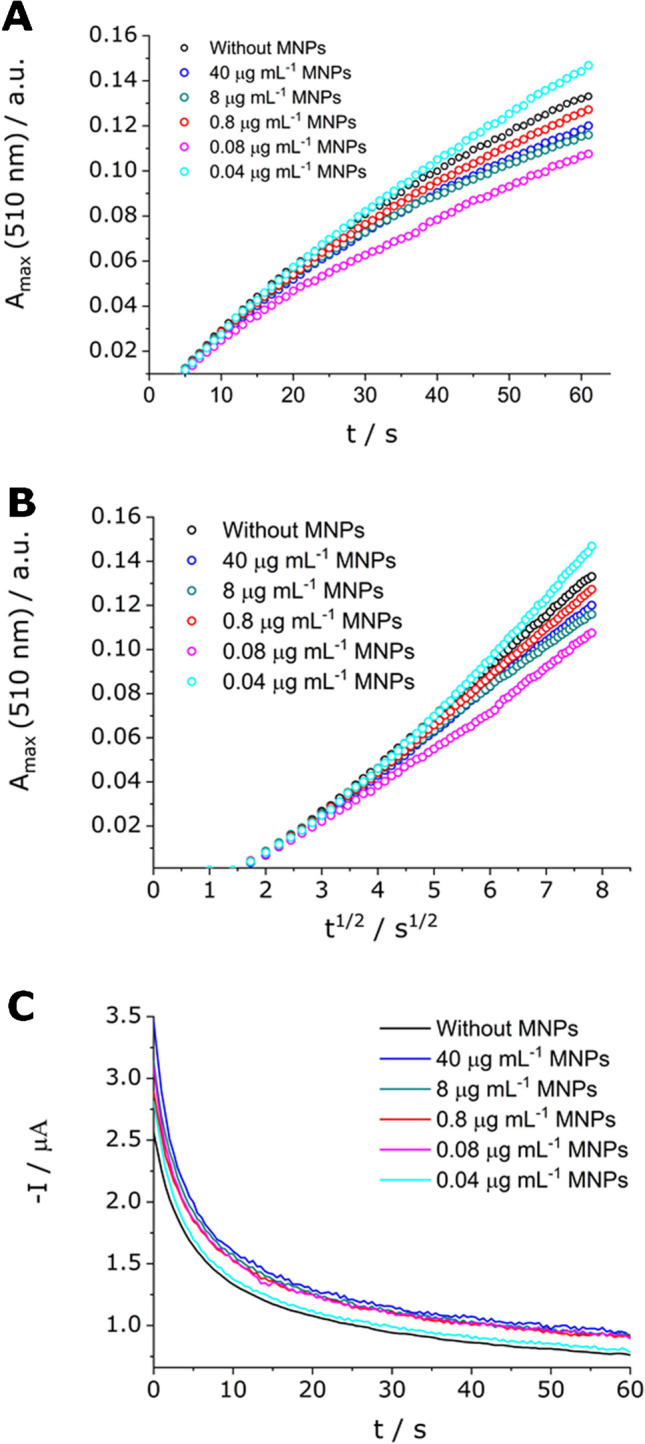
Effect of the concentration of cobalt ferrite MNPs in UV–Vis SEC measurements for solutions containing 25 mmol L^−1^ of OP and 0.3 mmol L^−1^ of Fe(III) without CoFe_2_O_4_ MNPs (black) and with 40 (blue), 8 (dark cyan), 0.8 (red), 0.08 (pink), and 0.04 (cyan) µg mL^−1^ of MNPs at a fixed potential and scan rate of − 0.2 V and 1000 rpm, respectively. **A** *A*_max_ (510 nm) as a function of *t*, **B** *A*_max_ (510 nm) as a function of *t*^1/2^, and **C** –*I* as a function of *t*

Finally, the optimization of CoFe_2_O_4_ MNP concentration was carried out. As shown in Fig. 5C, the addition of MNPs causes an increase in the current produced by the electrochemical process as compared to the situation without MNPs (black solid line), which agrees with previously explained results. Regarding the optical response, the solution with 0.04 µg mL^−1^ of MNPs (Fig. 5A and B, cyan dots) showed a higher optical signal in comparison to the solution without MNPs (Fig. 5A and B, black dots). Likely, the solution containing 0.8 µg mL^−1^ of cobalt ferrite MNPs shows a similar optical response to the solution without MNPs (Fig. 5A and B, red dots). Consequently, these concentrations were chosen as the optimal to carry out the study of the effect of nanostirring in the calibration plots.

### Study of the effect of nanostirring in the calibration plots of SEC experiments

Once the experimental conditions for SEC with nanostirring were optimized, calibration plots based on the slopes of absorbance versus time were constructed using various concentrations of Fe(III). This was done to compare the analytical performance of the new methodology against that obtained in the absence of stirring, as previously evaluated [11].

As shown in Figs. 4 and 5, the absorbance signal under nanostirring conditions does not consistently exhibit a linear dependence on time. Deviations from linearity are particularly noticeable at the beginning of the measurement, probably due to a transition from a pure diffusion regime to a convective diffusion regime. Additional deviations can be seen at longer times, possibly as a result of the instability of convective transport. In between these sections, a well-defined linear segment is observed, which is especially broad under the optimized experimental conditions. This linear portion was selected for slope determination, and these slopes were subsequently used to generate the calibration plots.

**Fig. 6:**
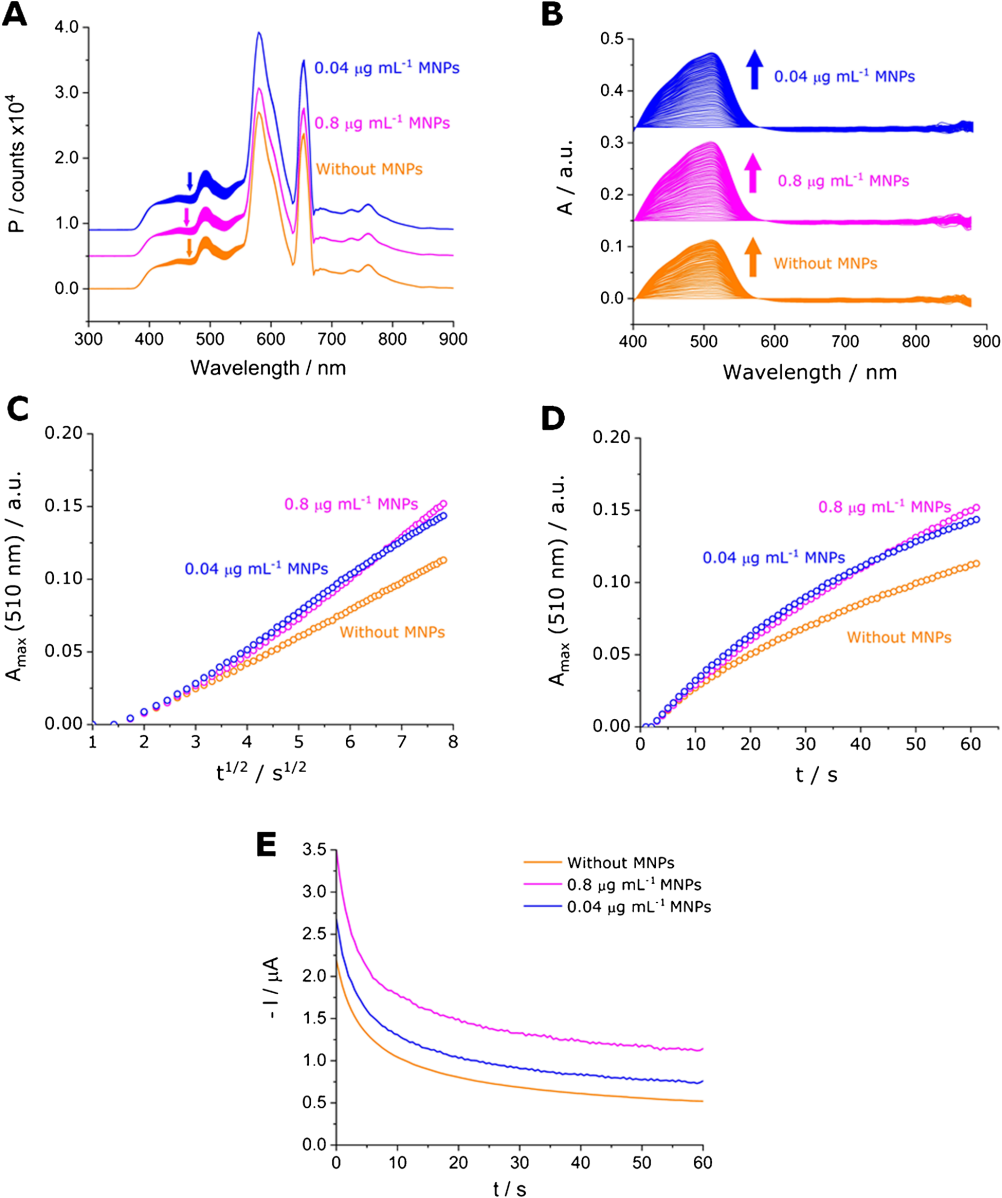
SEC signals obtained in the optimal optical and electrochemical conditions. Comparison between solutions containing 25 mmol L^−1^ of OP and 0.3 mmol L^−1^ of Fe(III) without MNPs (orange color) and with 0.8 (magenta color) and 0.04 (blue color) µg mL^−1^ at 1000 rpm when a fixed potential of − 0.2 was applied. **A** Counts as a function of wavelength, **B** absorbance as a function of wavelength, **C** maximum absorbance at 510 nm as a function of the square root of time, **D** maximum absorbance at 510 nm as a function of time, and **E** negative current as a function of time. In panels **A** and **B**, orange, magenta, and blue arrows indicate the evolution of the optical signal and magenta and blue spectra were shifted along the *y*-axis for comparison purposes

The improvements of introducing MNPs in the solution to perform SEC measurements are displayed in Fig. 6. The presence of low concentrations of MNPs (0.8 and 0.04 µg mL^−1^) in a solution of 0.3 mmol L^−1^ implies an increase in the optical signal (Fig. 6A, B, C, and D, magenta and blue colors) compared to a solution without MNPs (orange color). Moreover, the solutions with nanostirring (Fig. 6E) display a better electrochemical signal compared to still system.

It is important to recall that under a diffusion-controlled regime, Eq. (3) is applicable, predicting a linear relationship between absorbance (*A*) and the square root of time (*t*^*1/2*^). On the contrary, under a convective diffusion regime; where mass transport is enhanced by stirring; Eq. (2) becomes relevant, predicting a linear relationship between absorbance and time (*t*).

3 $$A= \frac{2 {D}^{1/2}\varepsilon }{{\pi }^{1/2}} {c}_{\text{ox}}^{*} {t}^{1/2}$$

**Fig. 7:**
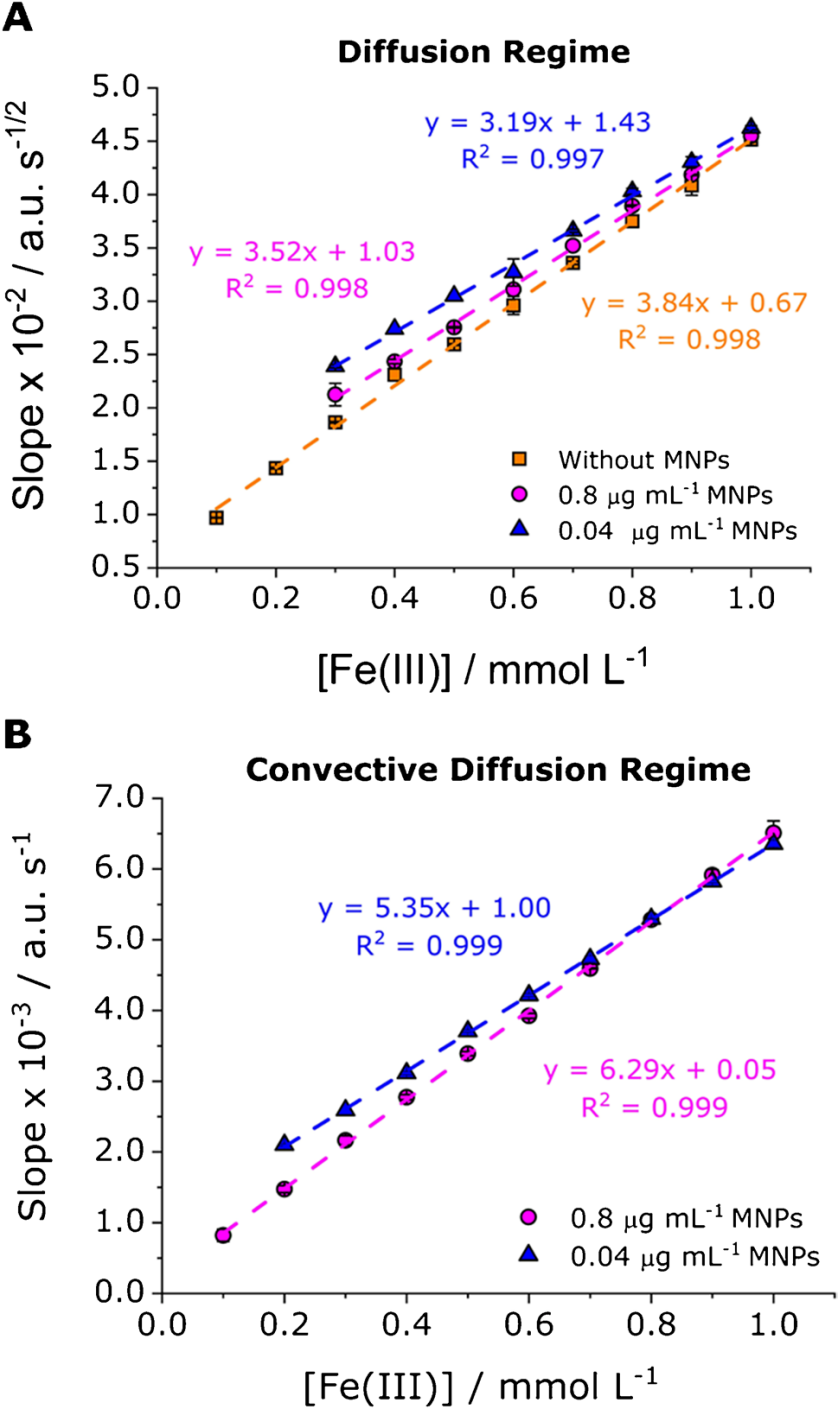
Calibration curves obtained from the UV–Vis spectrum of the SEC measurements: **A** slopes of the *A*_max_ (at 510 nm) vs.* t*.^1/2^ plot as a function of the concentration of Fe(III) and **B** slopes of the *A*_max_ (at 510 nm) vs.* t* plot as a function of the concentration of Fe(III)

Figure 7 and Table 1 summarize the calibration curves and associated analytical parameters obtained for Fe(III) solutions considering three different conditions: (i) without MNPs, (ii) and (iii) with MNPs at concentrations of 0.04 and 0.8 µg mL⁻^1^, respectively, all stirred at 1000 rpm. In the absence of stirring, mass transport during the electrochemical process is driven purely by diffusion. Accordingly, as predicted by Eq. 3, the “slope of slopes” for *A*_max_ (at 510 nm) was obtained assuming linearity concerning *t*^*1/2*^ (see Fig. 7B).

**Table 1:** Calibration parameters resulting from data acquired from SEC experiments in different conditions. Linear ranges are expressed from LOQ to the end of linearity

	Diffusion regime				Convective diffusion regime			
	CA slope *A*_max_ vs. *t*^1/2^				CA slope *A*_max_ vs. *t*			
	Linear range (mmol L^−1^)	Sensitivity × 10^−2^ (a.u. s^−1/2^ mmol^−1^ L)	LOD × 10^−2^ (mmol L^−1^)	*R*^2^	Linear range (mmol L^−1^)	Sensitivity × 10^−3^ (a.u. s^−1^ mmol^−1^ L)	LOD × 10^−2^ (mmol L^−1^)	*R*^2^
Fe(III) alone	0.09–1.00	3.84 (0.06)	2.77	0.998	NA	NA	NA	NA
Fe(III) + 0.8 μg mL^−1^ MNPs	0.10–1.00	3.52 (0.05)	3.08	0.998	0.04–1.00	6.29 (0.05)	1.34	0.999
Fe(III) + 0.04 μg mL^−1^ MNPs	0.13–1.00	3.19 (0.06)	3.96	0.997	0.03–1.00	5.35 (0.03)	1.05	0.999

Standard deviation is indicated in parentheses

In contrast, for nanostirred solutions, two transport regimes are evaluated. Initially, the system is considered to behave as a diffusion-controlled regime accordingly to Eq. 3 (Fig. 7A). Then, if a convective diffusion regime is applied, the “slope of slopes” for *A*_max_ (at 510 nm) was calculated as a function of time (Eq. 2) as shown in Fig. 7B. Under convective diffusion, LOD were lower and the sensitivity values were higher compared to the unstirred system. Specifically, LODs of 1.05 × 10⁻^2^ mmol·L⁻^1^ and 1.34 × 10⁻^2^ mmol·L⁻^1^ were obtained for solutions containing 0.04 and 0.8 µg·mL⁻^1^ of CoFe_2_O_4_ MNPs, respectively. These values represent a threefold and twofold enhancement, respectively, compared to the LOD obtained under diffusion-controlled conditions for the solution without MNPs (2.77 × 10⁻^2^ mmol·L⁻^1^). Additionally, the intercepts of the calibration curves obtained under nanostirring (Fig. 7B) were closer to zero than those derived from the *t*^*1/2*^-based plots (Fig. 7A), supporting the prevalence of convective diffusion. Remarkably, when assuming a diffusion-controlled regime for the nanostirred system, LOD and sensitivity values shown in Table 1 were worse in comparison to the ones obtained in still conditions. This fact suggests that a diffusion-controlled regime could not be assumed when CoFe_2_O_4_ MNPs induce nanostirring.

Overall, these results show that in the presence of nanostirring, mass transport in the electrochemical process is primarily caused by convective diffusion. This regime enhances the analytical performance of the method compared to the diffusion-driven case. The nanostirring effect on the electrochemical process is visually demonstrated in Video S3. The video shows how stirring induced by CoFe_2_O_4_ MNPs under an external magnetic field facilitates the removal of the red-colored Fe(II)-OP species formed during electrochemical reduction. This behavior indicates solution regeneration near the working electrode surface and a consequent increase in Fe(II)-OP generation.

This regime could also be explained by Peclet numbers in the range of 1 to 10 [41]. The rotational and translational motion of the MNPs, as observed by optical microscopy, reflects the combined influence of moderate magnetic torques, viscous drag, hydrodynamic interactions, and partial aggregation at higher MNP concentrations. Altogether, these factors limit the effective nanoparticle motion, leading to Peclet values consistent with mixed convective-diffusive transport. Similar behaviors have been described in magnetically actuated colloidal systems, where translational drift and rotational motion exhibit a phase lag relative to the rotating magnetic field due to hydrodynamic coupling and confinement effects [33, 42, 43]. Despite the external field rotating at 1000 rpm, the actual movement of the MNPs is significantly attenuated, producing low translational velocities that still enable sufficient fluid renewal to support a convective-diffusive regime without fully becoming a convection-dominated transport. This confirms that nanostirring operates within a defined operational range, where the moderate enhancement of mass transport contributes to improved SEC signals at optimal MNPs concentrations and field rotation rates.

## Conclusions

Magnetic nanostirring, as a proof of concept, appears to be a promising tool to enhance mass transport in tiny spaces. In SEC measurements, the use of cobalt ferrite MNPs has shown to be very effective in changing the mass transport regime from planar diffusion to convective diffusion. However, this strategy suffers from agglomeration of MNPs, which causes high currents in chronoamperometric plots, but with increased noise, and significant problems in absorption measurements due to light scattering. It was determined that the use of CTAB as a surfactant agent avoids the agglomeration of MNPs when a magnetic field is applied. Moreover, the use of lower concentrations of MNPs provides an improvement of optical measurements, when the convective diffusion regime is considered, more reproducible than in the case of using higher MNPs concentrations and an improvement in the LOD and sensitivity in the electrochemical Fe(III)/Fe(II)-OP system. Finally, the proposed approach based on the application of magnetic nanostirrers for SEC measurements could easily be extended to other analytical applications like miniaturized sensors, colorimetric assays, lab-on-a-chip microfluidics, in which a confined and reduced volume is required, and where mass transport limitations could compromise analytical performance. This depends on possible future optimization in terms of MNPs design (e.g., different shapes and compositions), more precise magnetic field control and assembly of other optical–electrochemical configurations (e.g., fluorescent-based).

## Supplementary Information

Below is the link to the electronic supplementary material.

Supplementary file1 (DOCX 1.46 MB)

Supplementary file2 (MP4 29.1 MB)

Supplementary file3 (MP4 18.2 MB)

Supplementary file4 (MP4 20.6 MB)

## Data Availability

No datasets were generated or analysed during the current study.
