# Supplementary material for: Cobalt ferrite magnetic nanoparticles as stirring actuators to improve UV–Vis spectroelectrochemical measurements in normal reflection mode
Source: Mikrochim Acta. 2025 Aug 1;192(8):546. doi: 10.1007/s00604-025-07351-2 (PMC12316778; doi:10.1007/s00604-025-07351-2)
Supplement: Supplementary file 1 — Supplementary file1 (DOCX 1.46 MB) [file 604_2025_7351_MOESM1_ESM.docx]

**Supplementary Information for**

**Cobalt ferrite magnetic nanoparticles as stirring actuators to improve UV-vis spectroelectrochemical measurements in normal reflection mode**

Alessandra Cutillo-Foraster^1^, Nurhayat Özbek^2^, Lluís Otero-de Muller^1^, Julio Bastos-Arrieta^1,3*^, Núria Serrano^1,3^, José Manuel Díaz-Cruz^1,3^

^1^Department of Chemical Engineering and Analytical Chemistry, Universitat de Barcelona (UB), Martí i Franqués 1-11, 08028 Barcelona, Spain,

^2^Department of Chemistry, Faculty of Sciences, Karadeniz Technical University, Trabzon 61080, Turkey

^3^Water Research Institute (IdRA), University of Barcelona (UB), Martí i Franquès 1-11, 08028-Barcelona, Spain

*Corresponding author to whom correspondence should be addressed: e-mail: [julio.bastos@ub.edu](mailto:julio.bastos@ub.edu),

**Discussion on spectroelectrochemical measurements**

In a previous study [1], the model system Fe(III) + ortho-phenanthroline (OP) was submitted to chronoamperometric measurements by using a commercial screen-printed carbon electrode (SPCE) and a reflection probe attached to the commercial cell DRP-REFLECELL 70055 (Fig. 1 A to C). Then, a fixed potential was applied to promote the reduction of the 1:3 Fe(III)-OP complex, not especially colored, to the 1:3 Fe(II)-OP complex, which produces an intense red color because it strongly absorbs at a wavelength close to 510 nm. Some electrochemical considerations on the evolution of the cathodic current (I) and the absorbance (A) with time (t) predicted a linear relationship of these parameters with respect to t^-1/2^ and t^1/2^, respectively [1, 2]:

$I= -\frac{nFSD^{1/2}}{\pi^{1/2}} c_{ox}^{*} t^{-1/2}$ (Eq. S1)

$A= \frac{2 D^{1/2}\varepsilon}{\pi^{1/2}} c_{ox}^{*} t^{1/2}$ (Eq. S2)

where n is the number of electrons of the electrochemical reduction, F is the Faraday constant, S is the electrode surface, D and C_ox_^*^ are the diffusion coefficient and the bulk concentration of the oxidized species, respectively, and ε is the molar absorptivity of the reduced species. Another important parameter is the flux (J) of oxidized species towards the solution, which is proportional to the current:

$J=-\frac{I}{nFS}= \frac{D^{1/2}}{\pi^{1/2}} c_{ox}^{*} t^{-1/2}$ (Eq. S3)

Indeed, the integral of the flux with respect to time is proportional to the concentration of reduced species and, hence, to the absorbance, which explains the proportionality between A and t^1/2^ shown in Eqn. S2 (the integral of t^-1/2^ concerning t is 2 t^1/2^).

The experiments carried out in [1] confirmed the applicability of the equations above to the experimental setup based on the commercial DRP-REFLECELL 70055. Moreover, they showed that the slope of the A vs. t^1/2^ plot, according to Eqn. S2, was a valuable analytical parameter to determine the concentration of Fe(III):

$slope= \frac{2 D^{1/2}\varepsilon}{\pi^{1/2}} c_{ox}^{*}=k [Fe\left( III \right)]$ (Eq. S4)

where k is a constant that include the terms $\frac{2 D^{1/2}\varepsilon}{\pi^{1/2}}$ of Eqn.4.

**Comparison between the working electrode surface of a SPCE and SPAgE**


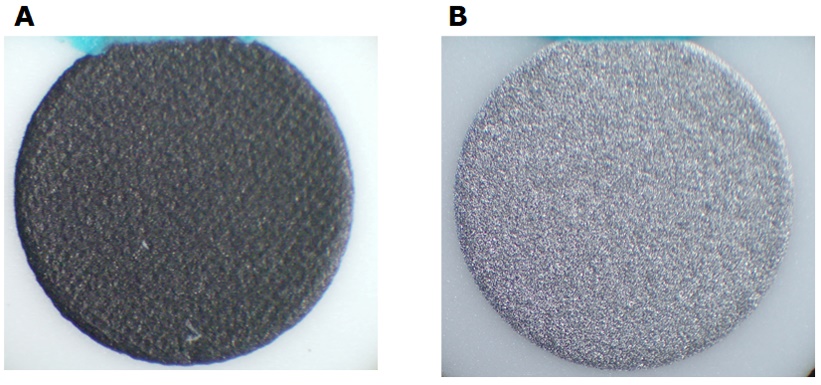
The Fig. S1 compares a microscopic image of the surface of the working electrode of a SPCE (Fig. S1 A) with the one of the SPAgE (Fig. S1 B). These images show that the surface reflectance in the SPAgE is higher than in the SPCE, for that reason, an electrode of SPAgE was chose to registered the videos S1, S2 and S3.

**Fig. S1** Comparison of the working electrode surface. Microscopic images of the surface of an **A)** SPCE and **B)** SPAgE.

**Absorbance spectra of OP complexes of Fe(II) and Fe(III)**


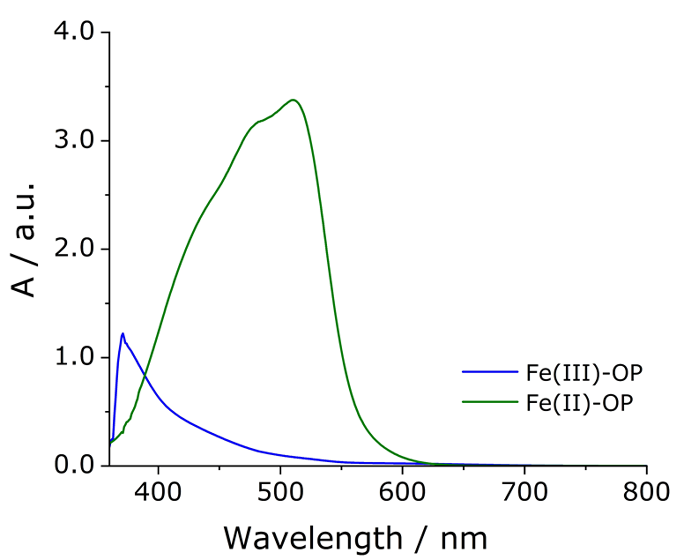
Fig. S2 shows the absorbance spectra registered for Fe(III)-OP and Fe(II)-OP complexes in which Fe(II)-OP complex exhibited a strong absorbance near the wavelength of 510 nm. In contrast, in this region, the complex Fe(III)-OP did not show any absorbance peak. This leads to confirm that the absorbance registered in the SEC measurements only belonged to Fe(II)-OP.

**Fig. S2** Comparison absorbance spectrum obtained for solutions containing 25 mmol L^-1^ of OP in acetate buffer solution (pH = 4.50) with 0.3 mmol L^-1^ of Fe(III) (blue color) and 0.3 mmol L^-1^ of Fe(II) (green color).


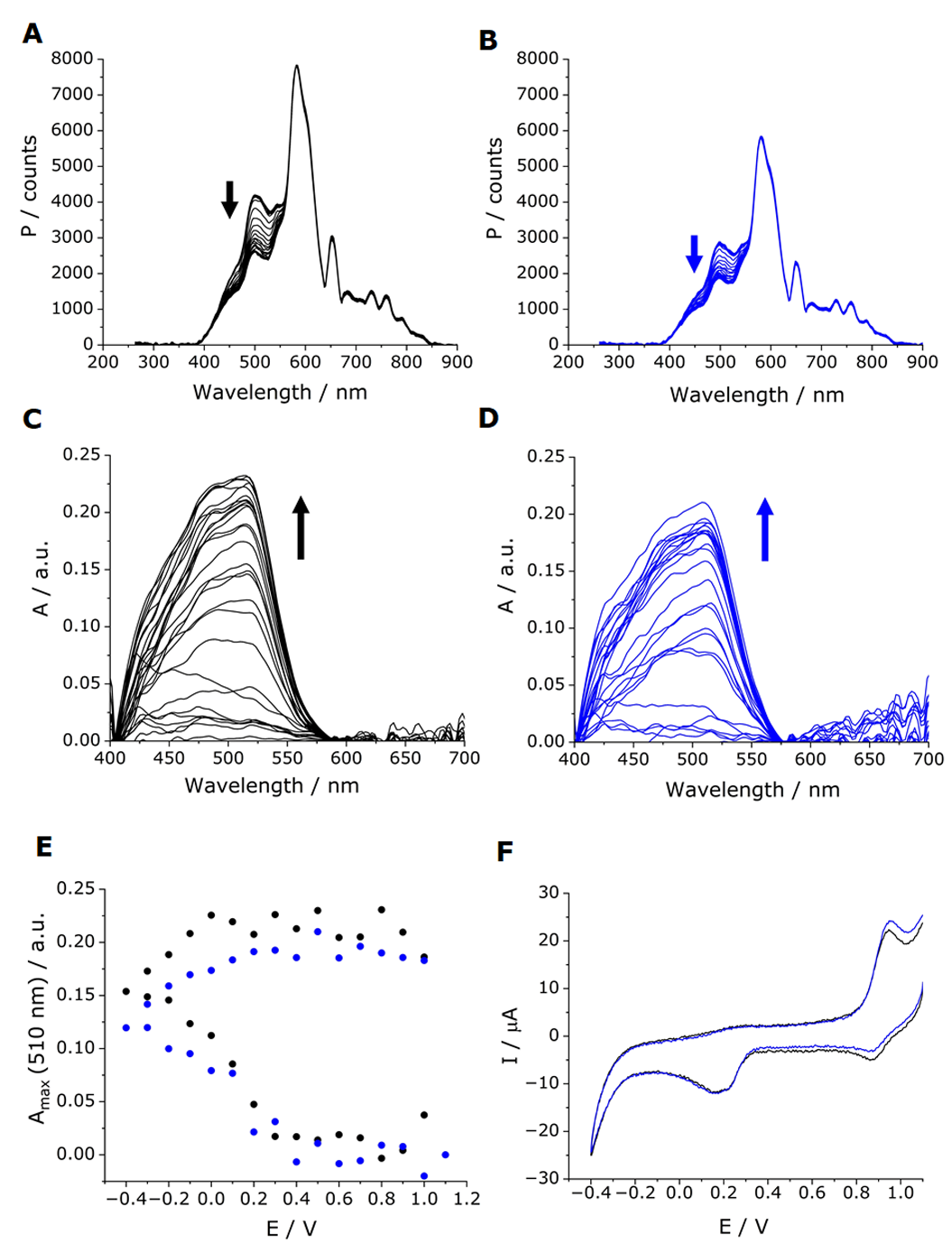
**Preliminary study of the effect of high concentration of CoFe_2_O_4_ MNPs in SEC measurements**

**Fig. S3** Comparison between the SEC signals obtained for a solution 25 mmol L^-1^ in OP and 1.0 mmol L^-1^ in Fe(III) in the absence of MNPs (black color) and in the presence of 300 µg mL^-1^ of MNPs (blue color), showing the respective reflection spectra in **A)** and **B)** counts and in **C)** and **D)** absorbance, **E)** the maximum absorbance (at 510 nm) as a function of the applied potential (from 1.1 V to -0.4 V and then to 1.05 V) and **F)** the cyclic voltammograms (from 1.1 V to -0.4 V and then to 1.05 V). Black and blue arrows in Fig.S1 **A)**, **B)**, **C)** and **D)** show the evolution of the optical signal.

**Study of the electrocatalytic effect of CoFe_2_O_4_ MNPs in the electrochemical signal**


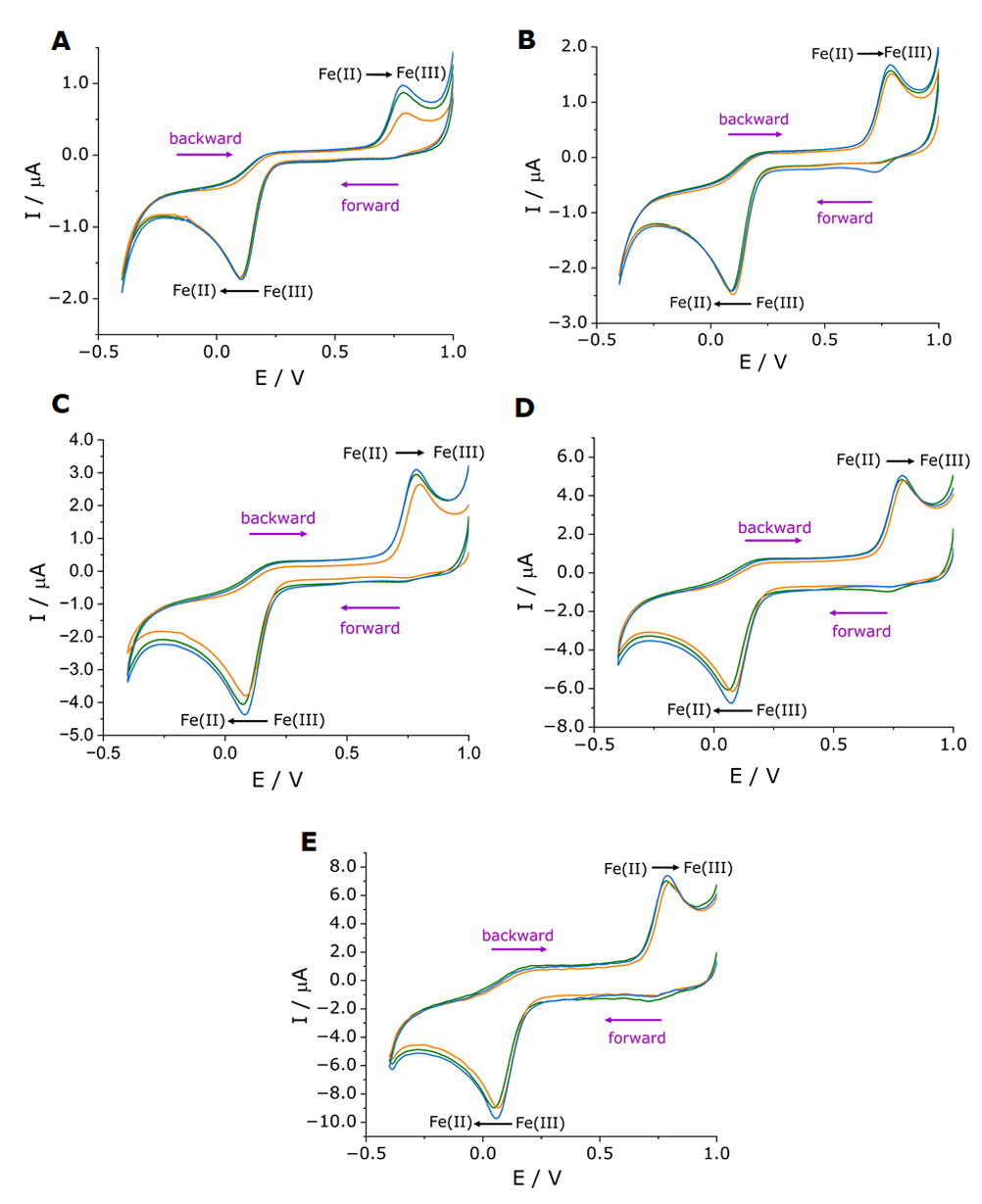
The electrocatalytic effect of MNPs on the electrochemical behavior of Fe(III)/Fe(II)-OP system was studied for a solution of 0.3 mmol L^-1^ without MNPs and with MNPs (40 and 0.8 µg mL^-1^) at different scan rates. It was seen that MNPs do not have an electrocatalytic effect around the peak at ca. 0.2 V that belongs to the reduction reaction of Fe(III) to Fe(II), which is the electrochemical process studied in this work.

**Fig. S4** Comparison CV (from 1.0 V to -0.4 V and then 1.0 V) obtained for solutions without CoFe_2_O_4_ MNPs (green color) and with 40 (orange color) and 0.8 (blue color) µg mL^-1^ CoFe_2_O_4_ MNPs at different scan rates **A)** 10, **B)** 20, **C)** 50, **D)** 100 and **E)** 200 mV s^-1^.

**
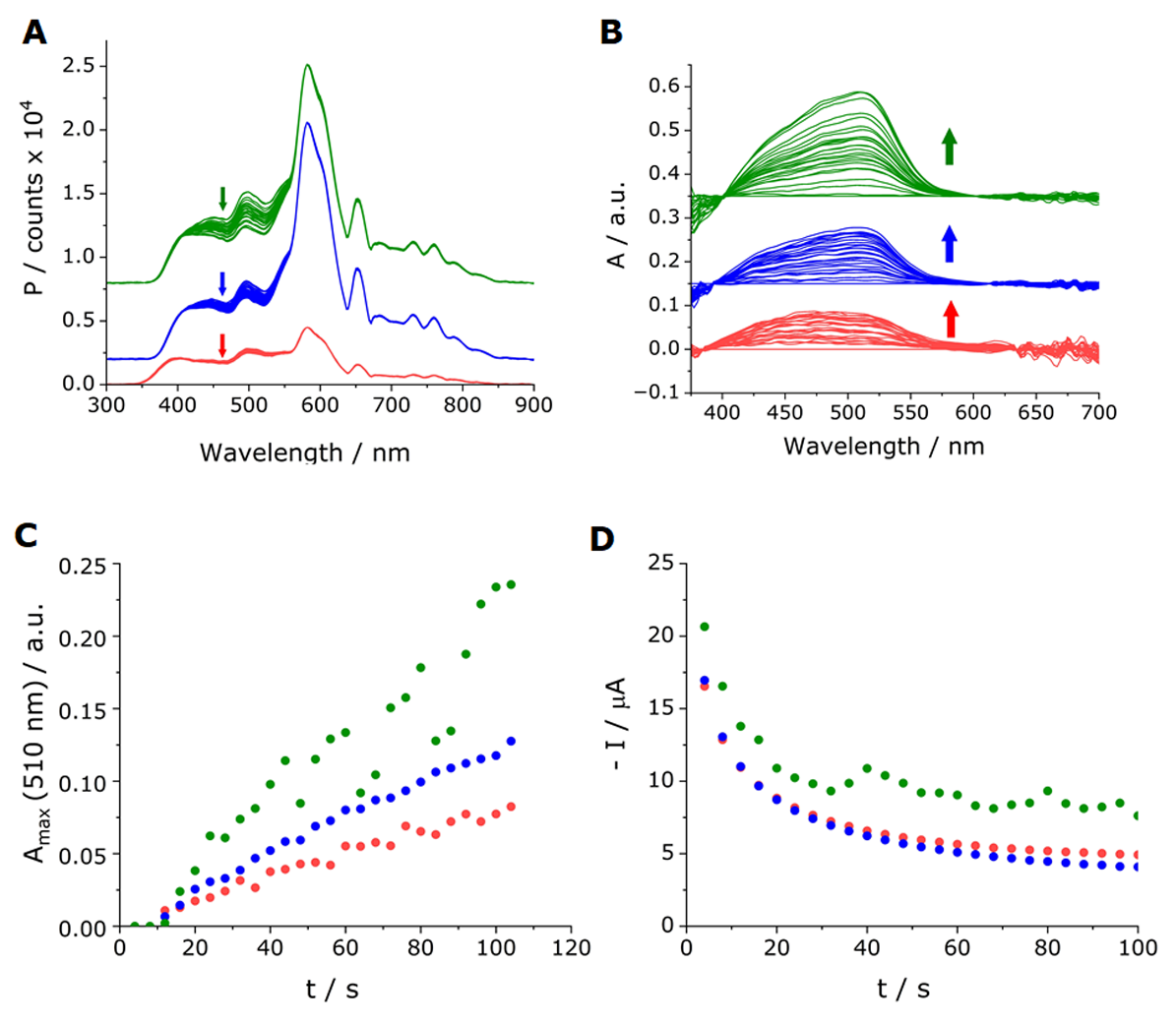
Study of the effect of CoFe_2_O_4_ MNPs with and without stirring in SEC measurements**

**Fig. S5** Comparison between the SEC signals obtained for a solution 25 mmol L^-1^ in OP and 0.5 mmol L^-1^ in Fe(III) in the presence of 400 µg mL^-1^ of MNPs, showing the corresponding reflection **A)** spectra in counts and **B)** in absorbance, **C)** the maximum absorbance (at 510 nm) as a function of the time and **D)** the chronoamperograms at a fixed potential of -0.20 V for suitable experiments in the absence of stirring (blue color) and with solution stirring (green color), and also for one containing MNPs (red color) without stirring, where the counts are too low. Red, blue and green arrows in Fig.S1 **A** and **B** show the evolution of the optical signal and blue and green spectra have been shifted along the y axis for comparison purposes.

**Description of videos in the supplementary information**

**Supplementary Video S1**

The Video S1 shows the effect of increasing the stirring rate on the movement of MNPs for a solution containing 400 µg mL^-1^ in acetate buffer solution at different stirring rates (250, 500, 750, 1000, 1500, 2000 and 2500 rpm). When the magnetic stirrer is on, the agglomerates show a clockwise rotation and a counter-clockwise translation, with increased speed for the particles that are far from the center of the electrode/magnetic stirrer. Moreover, raising the stirring rate causes an increase in both rotation and translation rates. More precisely, the translation movement prevails at stirring rates above 1000 rpm.

**Supplementary Video S2**

The Video S2 shows the effect of introduction CTAB, which is a surfactant agent, in solutions containing different concentration of CoFe_2_O_4_ MNPs. More precisely, it compares a solution containing 400 µg mL^-1^ MNPs in an acetate buffer solution with two solutions with 400 and 40 µg mL^-1^ MNPs were both of them were suspended in a solution with 1 mmol L^-1^ CTAB. All solutions were stirred at 1000 rpm. Video S2 shows that the addition of CTAB minimizes the formation of large agglomerations, which may be explained due to MNPs were stabilized in aqueous solution by decreasing their hydrophobic behavior through interaction between the non-polar chain of CTAB and MNPs surface [3].

**Supplementary Video S3**

The Video S3 shows the effect of nanostirring in the electrochemical reduction of Fe(III) in a SPAgE at a fixed potential of -0.2 V during 60 s by comparing a solution with a concentration of 1.0 mmol L^-1^ of Fe(III) without and with 40 µg mL^-1^ of CoFe_2_O_4_ MNPs at 1000 rpm. Each solution contains 25 mmol L^-1^ of OP in acetate buffer. The video shows how stirring induced by CoFe_2_O_4_ MNPs under an external magnetic field facilitates the removal of the red-coloured Fe(II)-OP species formed during electrochemical reduction. This behaviour indicates solution regeneration near the working electrode surface and a consequent increase in Fe(II)-OP generation.

**References**:

1. Sirin Ustabasi G, Bastos-Arrieta J, Pérez-Ràfols C, et al (2022) Considerations on the use of spectroelectrochemistry in reflection mode for quantitative analysis: Study of the Fe(III)/Fe(II) – orthophenanthroline system. Microchemical Journal 181:1–10. https://doi.org/10.1016/j.microc.2022.107678

2. Bard AJ, Faulkner LR (2001) Spectroelectrochemistry and other coupled characterization methods. In: Electrochemical Methods: Fundamentals and Applications, 2nd Edn. John Wiley

3. Ramos Guivar JA, Sanches EA, Magon CJ, Ramos Fernandes EG (2015) Preparation and characterization of cetyltrimethylammonium bromide (CTAB)-stabilized Fe3O4 nanoparticles for electrochemistry detection of citric acid. Journal of Electroanalytical Chemistry 755:158–166. https://doi.org/10.1016/j.jelechem.2015.07.036
